# Cryogenic Dielectric Metasurface‐Integrated Superconducting Nanowire Single‐Photon Detectors

**DOI:** 10.1002/nap2.70172

**Published:** 2026-06-28

**Authors:** Amir Targholizadeh, Grigoriy Y. Nikulin, Pankaj K. Jha

**Affiliations:** ^1^ Quantum Technology Laboratory 〈Q|T|L〉 Department of Electrical Engineering and Computer Science Syracuse University Syracuse New York USA; ^2^ Institute for Quantum & Information Sciences Syracuse University Syracuse New York USA

## Abstract

Over the past decade, multielement superconducting nanowire single‐photon detectors (SNSPDs) have emerged as the leading single‐photon detection technology due to their exceptional system detection efficiency (SDE), ultrahigh timing precision, and negligible dark counts. However, achieving these performances with a *single‐element* SNSPD has been an outstanding challenge due to a fundamental trade‐off: A large active area is necessary for high SDE, whereas a smaller area is crucial for higher photon count rates, lower dark counts, and lower jitter. Here, we introduce a dielectric cryogenic metalens integrated with a single‐element SNSPD to achieve high SDE with a smaller active area. We report a ∼77‐fold enhancement in the SDE of a metalensed SNSPD with a 5 μm × 5 μm active area, compared with an identical reference SNSPD without a metalens, using a moderately sized 60 μm × 60 μm metalens. Next, we theoretically demonstrate polarization‐resolved photodetection at the telecommunication wavelength using a bifunctional metalens. We achieve a cross‐detection probability of < 2 % between metalensed SNSPDs spaced at 10 μm under illumination with *x*‐ and *y*‐polarized light. Integrating a multifunctional cryogenic metasurface with the state‐of‐the‐art SNSPDs may enable novel capabilities with reduced size, weight, power, cost, and cooling (SWaP‐$C^{2}$) requirements.

## Graphical abstract

Superconducting nanowire single‐photon detectors (SNSPDs) are the leading technology for single‐photon detection because of their near‐unity system detection efficiency, ultrahigh timing precision, and very low dark counts. However, achieving these performance metrics with single element, small‐active‐area SNSPDs has been an outstanding challenge. Here, we leveraged the exceptional light‐molding capabilities of a dielectric metalens to develop a miniaturized single element SNSPD that offers high system detection efficiency, sensitivity, and low noise.

## 1 Introduction

Over the last decade, superconducting nanowire single‐photon detectors (SNSPDs) have emerged as a leading single‐photon detection technology owing to near‐unity system detection efficiency (SDE) [1, 2], ∼ GHz photon count rate (PCR) [3, 4], dark count rate (DCR) below $10^{-5}$ counts/s [5], and ∼ ps timing resolution [6]. This remarkable progress has enabled high‐rate (1.3 Gb $s^{-1}$) [7] and long‐distance quantum key distribution (404 km [8]), long‐distance quantum communications (> 1200 km) [9], among others. However, optimizing these device metrics simultaneously within a *single‐element* SNSPD remains an outstanding challenge due to the inherent trade‐offs among them. A typical single‐element SNSPD consists of a thin (∼5–10 nm), narrow (∼100 nm), and long wire (a few hundred micrometers) made of a low‐temperature superconductor, such as niobium nitride (NbN), niobium titanium nitride (NbTiN), or tungsten silicide (WSi), coiled into a meander pattern covering an active area large enough to capture light from a coupled single‐mode optical fiber and accommodate slight fiber misalignment [10, 11, 12, 13, 14]. Achieving high PCR, low DCR, and low timing jitter requires a shorter nanowire. However, as the nanowire length decreases, the active area shrinks, which, in turn, reduces the SDE critical for “photon‐starved” applications [15, 16, 17, 18]. A straightforward approach to significantly reduce the nanowire length without compromising the active area is to implement an interleaved design [19]. This method substitutes a single elongated nanowire with an array of multiple shorter nanowires sharing the same active area [20, 21, 22, 23]. However, as the number of interleaved nanowires increases within a given active area, cross talk between them due to electrical or thermal coupling becomes detrimental. Additionally, because each nanowire operates independently, it needs separate biasing, readout circuits, and other components, which can significantly increase the complexity of the readout system and the thermal load of the detector, creating challenges for scalability [24, 25, 26]. Another approach is to efficiently expand the collection area with a conventional lens while maintaining a smaller active area [27]. This approach eliminates the need for a single‐mode fiber to couple light to the SNSPD, thereby overcoming the limitation imposed by the fiber's core diameter on the minimum active area in conventional fiber‐coupled SNSPDs. Furthermore, free‐space‐coupled SNSPDs are especially promising for long‐wave infrared (LWIR) applications, where single‐mode fibers are more rigid and fragile. Recently, a single‐element and lensed NbN SNSPD exhibited an SDE of 72% ± 3.7% at 1550 nm with sub‐0.1 Hz DCR and sub‐15 ps timing jitter [28]. However, the full potential of this approach remains unrealized because it relies on conventional optical elements that are large, bulky, and less congruent with integrated quantum photonic systems.

Here, we propose and demonstrate a long‐sought‐after solution for a miniaturized single‐element SNSPD with high SDE while preserving the benefits of a smaller active area. We harness the exceptional optical wavefront molding capabilities of an array of judiciously designed dielectric nanoantennas, also known as a metasurface, to serve as a flat lens that significantly enhances the effective collection area of a single‐element SNSPD, thereby enabling a higher SDE with a smaller active area. Furthermore, we designed a polarization beam‐splitting metalens to demonstrate polarization‐resolved single‐photon detection at 1550 nm using a pair of single‐element SNSPDs.

Using dielectric metasurfaces to miniaturize single‐element SNSPDs offers several advantages. First, metasurfaces enable simultaneous control of key light properties—phase, amplitude, and polarization—thereby providing a promising way to replace bulky optical components with flat, ultrathin, and lightweight CMOS‐compatible alternatives [29, 30, 31, 32, 33]. Second, metasurfaces offer flat optical components at LWIR wavelengths where larger‐core optical fibers necessitate larger active areas for SNSPDs and thus suffer from low PCR, high DCR, and poor timing jitter [34]. Third, a single metasurface can provide complex functionalities that would otherwise require a combination of several bulky conventional optical components [35, 36, 37, 38, 39]. This may enable novel capabilities in the state‐of‐the‐art SNSPDs with reduced size, weight, power, cost, and cooling (SWaP‐$C^{2}$) requirements.

## 2 Metalensed SNSPD

Figure 1 illustrates a schematic of a dielectric metalens that focuses an incoming x‐polarized light onto a meander‐shaped single‐element SNSPD aligned with the polarization direction. Unlike a conventional lens that employs refraction of light, a metalens [40, 41, 42] focuses light by shaping the incident wavefront through abrupt phase changes locally imparted by the nanoantennas. The metalens collects light over an area $A_{M}$ and focuses it onto the SNSPD's active area $A_{D}$, effectively increasing the light‐collection area from $A_{D}$ to $\eta_{c}A_{M}$ without requiring a larger active area. Here, the optical coupling efficiency $\eta_{c}$ is defined as the ratio of the intensity of light incident on the SNSPD's active area to that on the metalens. The SNSPD is composed of an 8 nm thick and 100 nm wide niobium titanium nitride (NbTiN) meander‐shaped nanowire with a 50% fill fraction and covers an active area $A_{D}$ = 5×5 $\mu m^{2}$, which is about an order of magnitude smaller than that of a typical SNSPD operating at a 1550‐nm wavelength [1]. Without loss of generality, we considered NbTiN as the superconducting material for photodetection, as various groups have experimentally demonstrated high efficiency, low timing jitter, low dark‐count rate, and high photon detection rates with NbTiN‐based SNSPDs [43, 44]. The NbTiN nanowires are integrated with a simple cavity structure, consisting of a ${\text{SiO}}_{2}$ spacer layer and a gold back‐reflector to optimize photoabsorption [45, 46]. The absorptance A of the nanowire is given by the following equation [46]:

(1) $$A=\frac{\int_{V}\omega \;I\left[\varepsilon \left(r\right)\right]\;|E\left(r\right){|}^{2}dV}{\frac{1}{\eta_{0}}\int_{S}|E_{\text{inc}}{|}^{2}\;dS},$$

where ω is the optical angular frequency, I[ε(r)] is the imaginary component of the NbTiN permittivity [47], $|E\left(r\right){|}^{2}$ is the time‐averaged local electric‐field intensity within the nanowires, $E_{\text{inc}}$ is the amplitude of the incident field, and $\eta_{0}$ is the impedance of free space. Our simulation shows that, without the cavity, the absorptance of an infinite NbTiN grating on a ${\text{SiO}}_{2}$ substrate is 23% for a plane wave at normal incidence, and the absorptance increases to > 92% with a cavity with a 233 nm thick ${\text{SiO}}_{2}$ layer (see Supporting Information S1: Section 1, Figure S1a).

**FIGURE 1:**
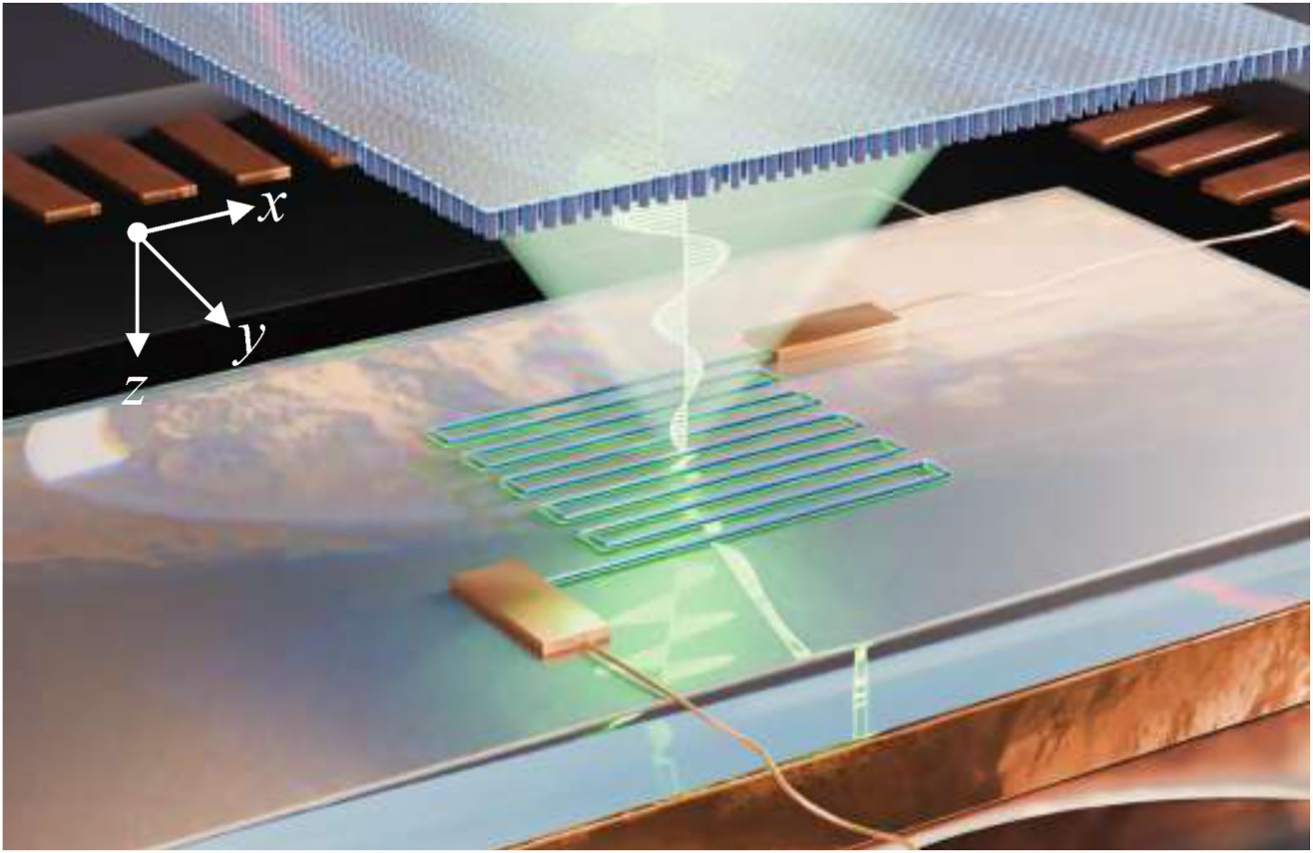
Metalensed single‐element SNSPD. Schematic illustration of a dielectric a‐Si metalens that effectively enhances the collection area of a miniaturized single‐element SNSPD by capturing incoming light over the metalens area $A_{M}$ and focusing it onto the active area $A_{D}$. The meander‐shaped SNSPD is integrated with an optical cavity to optimize the absorptance of the focused light. For a moderately sized 60μm × 60μm metalens, the system detection efficiency of the metalensed SNSPD is ∼77‐fold higher than that of an identical reference SNSPD without a metalens.

The phase profile across the metalens is given by the following equation [40, 41, 42]:

(2) $$\varphi \left(x,y\right)=-\frac{2\pi }{\lambda }\left(\sqrt{x^{2}+y^{2}+f^{2}}-f\right)$$

where λ is the wavelength in vacuum, and f is the focal length of the metalens. For the designed wavelength of 1550 nm, our metalens is composed of an array of amorphous silicon (a‐Si) cylindrical nanopillars (as shown in Figure 2a) of height H = 850 nm on a silicon dioxide (${\text{SiO}}_{2}$) substrate with a square lattice of pitch P = 600 nm. The refractive index of a‐Si at 5 K was taken from Ref. [48]. By changing the diameter of the nanopillars, the phase of the transmitted light is molded to span the entire 2π phase range. We mimicked the metalens profile with a set of eight different nanocylinder phase shifters, yielding a transmission efficiency of 86% for the metalens (see Supporting Information S1: Section 2, Figure S2a). To quantify the performance of our metalensed SNSPD, we used the quantitative description of the system detection efficiency (SDE) defined as $\eta_{\text{SDE}}=\eta_{c}\times \eta_{a}\times \eta_{r}$, where $\eta_{c},\eta_{a}$, and $\eta_{r}$ are the optical coupling efficiency, absorption efficiency, and registering efficiency, respectively [10]. Assuming that $\eta_{r}$ remains the same for a metalensed detector and a reference detector (without a metalens and operating in free space) and $\eta_{c}$ of the reference detector is unity, the enhancement factor in the SDE of a metalensed SNSPD (subscript *M*) compared to that of the reference detector (subscript *R*) can be defined as follows [49]:

(3) $$\frac{\eta_{\text{SDE},M}}{\eta_{\text{SDE},R}}=\left(\frac{\eta_{c,M}A_{M}}{A_{D}}\right)\frac{\eta_{a,M}}{\eta_{a,R}}$$

**FIGURE 2:**
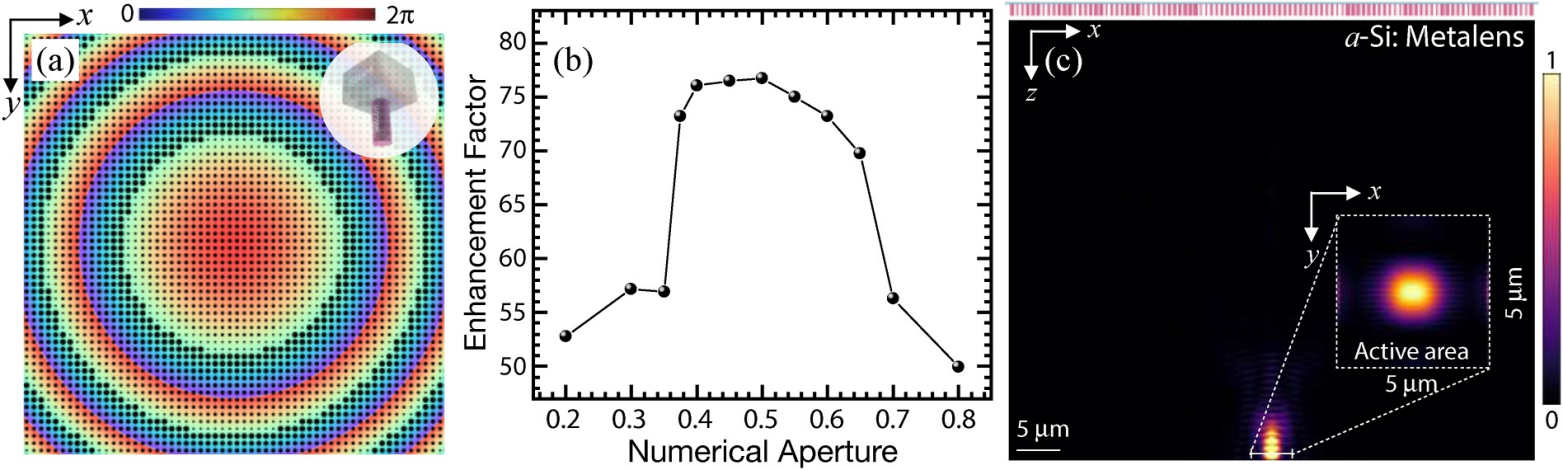
Metalensed NbTiN SNSPD SDE enhancement. (a) For the designed wavelength of 1550 nm, our metalens used a set of eight nanopillars with radii of 0.180 μm, 0.193 μm, 0.214 μm, 0.242 μm, 0.102 μm, 0.140 μm, 0.157 μm, and 0.168 μm corresponding to π/8,3π/8,5π/8,7π/8,9π/8,11π/8,13π/8,15π/8 phase shifts, respectively. The height of each nanopillar is set to 850 nm, and the pitch is 600 nm. (b) Plot of the enhancement factor of the system detection efficiency of the metalensed SNSPD defined as $\eta_{\text{SDE,}\;M}/\eta_{\text{SDE,}\;R}$ against the numerical aperture of the metalens of size $A_{M}$ = 60×60 $\mu m^{2}$. (c) Plot of intensity distribution of the metalens of NA = 0.5 and illuminated by an x‐polarized light in the x‐z plane. The inset shows the intensity distribution in the x‐y plane of the NbTiN nanowires.

Choosing an optimal numerical aperture (NA) of the metalens is crucial for a small detector area $A_{D}$ = $5\times 5\;\mu m^{2}$. Intuitively, a moderate‐to‐high‐NA metalens would be necessary to focus incoming light to a spot size (diameter) of less than 5μm. However, as the NA of a metalens increases, it exhibits a gradual decrease in focusing efficiency [50, 51], thereby lowering the coupling efficiency $\eta_{c,M}$. Furthermore, the components of the Poynting vector ($S_{x},S_{y},S_{z}$) redistribute in the focal plane, affecting the absorption efficiency $\eta_{a,M}$. Figure 2b shows a plot of the enhancement factor $\eta_{\text{SDE,}M}/\eta_{\text{SDE,}R}$ as a function of the NA of the metalens. For these simulations, we fixed the area of the metalens $A_{M}$ = 60×60 $\mu m^{2}$ (to keep the metalens footprint small and computationally light) and varied the focal length *f* from ∼ 147 μm to 22.5 μm. This range of focal lengths spanned NA values from 0.2 to 0.8. It is well known that a metalens exhibits an NA‐dependent focal length shift; that is, the simulated focal length ($f_{s}$) is different from the designed focal length (f) used in Equation (2) [52]. Therefore, for each NA, the detector is considered at the simulated focal length rather than the designed focal length (see Supporting Information S1: Section 3, Figure S3a). Our simulations show that a moderately sized metalens can provide an enhancement factor of ∼77 for NA = 0.5, corresponding to a simulated focal length of ∼52 μm. At this NA, our simulations show a coupling efficiency $\eta_{c,M}∼$ 0.66 (see Supporting Information S1: Section 4, Figure S4). The enhancement of SDE is sensitive to the alignment (position and orientation) of the SNSPD relative to the metalens. For the small‐active‐area SNSPD considered in this study, the enhancement factor drops by ∼ 1% if an optimally oriented detector is shifted laterally by ∼ 30 % of the size of our detector (see Supporting Information S1: Section 3, Figure S3b) from its ideal position. A further increase in the lateral misplacement, for instance, by ∼50 % of the detector size, results in a ∼ 37% drop in the enhancement factor. However, despite this drop in the enhancement factor, the SDE of a metalensed SNSPD is still ∼48‐fold higher than that of an identical reference SNSPD. Thus, achieving optimal performance in a metalensed SNSPD would require precise alignment and benefit from well‐developed nanofabrication technologies. Figure 2c shows the focusing effect of the metalens as shown by the field intensity distribution in the x‐z plane. The focal spot on the NbTiN nanowire (x‐y plane) is shown in the inset, and the full width at half maximum (FWHM) of the intensity profile along the x‐axis on the focal plane is 1.47 μm (see Supporting Information S1: Section 2, Figure S2b). In the inset of Figure 2c, the SNSPD's active area is highlighted by a dashed white square of length 5 μm. A tighter focal spot is useful for maintaining high SDE enhancement despite imperfect metalens performance due to fabrication imperfections, misalignment, etc. The tolerance can be further improved by increasing the SNSPD's active area, the size of the metalens, etc.

## 3 Multifunctional Metalensed SNSPD

Polarization is one of the fundamental properties of light, and its detection has diverse applications ranging from biomedical imaging and remote sensing to quantum key distribution and astronomy [53]. Conventional meander‐shaped SNSPDs are inherently polarization‐sensitive. Our simulations show that NbTiN nanowires, under plane‐wave illumination, show polarization dependence: Absorptance exceeds 92% for E‖ wire but drops to 45% for E⊥ wire (see Supporting Information S1: Section 1, Figure S1b). For circular polarization, the absorptance is ∼68%. The degree of polarization $C=\left(N_{\|}-N_{⊥}\right)/\left(N_{\|}+N_{⊥}\right)=0.34$ is low and thereby requires a polarization filter or a polarizer before detection. A unique feature of metasurfaces is the ability to encode more than one function in a single layer [54, 55, 56]. Furthermore, judiciously designed polarization‐dependent metasurfaces can induce novel quantum optical effects [57, 58, 59, 60, 61, 62]. Here, we designed a dielectric linear polarization beam splitter (LPBS) metalens integrated with a pair of mutually orthogonal NbTiN SNSPDs to achieve polarization‐resolved single‐photon detection at a telecommunication wavelength.

Figure 3a–c shows a schematic illustration of a dielectric LPBS metalens, which combines a metalens with a linear polarization beam splitter. Here, incident circularly polarized light is decomposed into its x‐ and y‐polarized components, which are then focused onto SNSPDs aligned with the x‐ and y‐axes, respectively. Similarly, if the LPBS metalens is illuminated with x‐polarized (y‐polarized) light, the light is focused on the x‐aligned (y‐aligned) SNSPD. Taking the metalens center as the origin (0, 0, 0), Equation (2) gives the phase profile for an on‐axis focus at (0,0,f). In our design, this phase is modified to produce two laterally displaced, cofocal spots at $\left(-x_{0},0,f\right)$ and $\left(x_{0},0,f\right)$, routing the x‐polarized component to the negative‐x focus and the y‐polarized component to the positive‐x focus. The corresponding aperture‐plane phases are $\varphi_{x,y}\left(x,y\right)=-\left(2\pi /\lambda \right)\left(\sqrt{{\left(x\pm x_{0}\right)}^{2}+y^{2}+f^{2}}-f\right)$ whose equiphase contours are circular and centered at $\left(-x_{0},0\right)$ for $\varphi_{x}$ and at $\left(x_{0},0\right)$ for $\varphi_{y}$; see Figure 3b,c. Both phase maps are realized on a single metasurface by exploiting form birefringence: Rectangular a‐Si nanobricks on a low‐index ${\text{SiO}}_{2}$ substrate exhibit different effective indices for the TE and TM modes, enabling independent phase control of orthogonal polarizations. At λ=1550nm, sweeping the in‐plane widths $\left(w_{x},w_{y}\right)$ yields full 0‐2π phase coverage for both polarizations with high transmission. We quantize each polarization’s phase to eight levels over 0‐2π in π/4 increments; the resulting 8×8 phase grid requires 64 phase‐pair states. We set the unit‐cell pitch to 500 nm and the nanobrick height to h=1960 nm, which is larger than that in typical 1550 nm metalenses [63], to increase modal separation and provide two independent phase degrees of freedom per unit cell. We precompute a library of 64 nanobrick geometries that map $\left(\varphi_{x},\varphi_{y}\right)$ to $\left(w_{x},w_{y}\right)$ with mean transmittance >90% (see Supporting Information S1: Section 5, Table S1 and Figure S5a).

**FIGURE 3:**
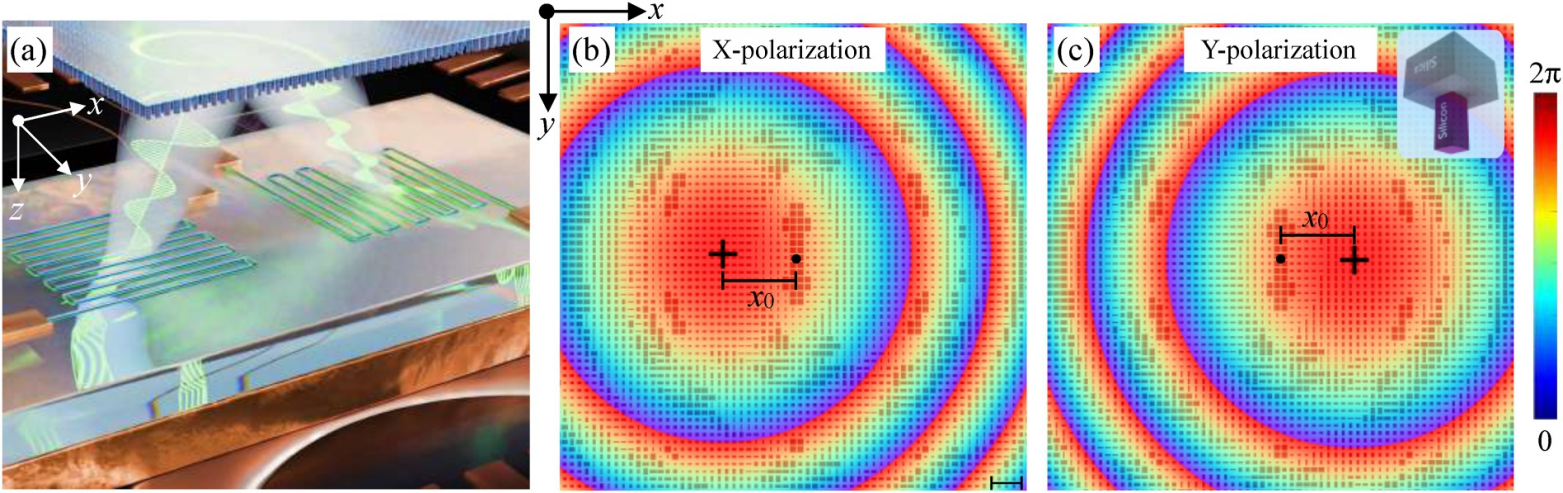
Bifunctional metalensed single‐element SNSPDs. (a) Schematic illustration of dielectric LPBS metalensed SNSPDs. An incident circularly polarized beam is decomposed into its x‐ and y‐polarized components, which are focused onto spatially separated meandered SNSPDs oriented along the x‐ and y‐axes, respectively. For linearly polarized input, an x‐polarized (y‐polarized) beam is focused onto the x‐oriented (y‐oriented) SNSPD. (b–c) The LPBS metalens design employs superimposed phase profiles corresponding to off‐axis focusing for the two orthogonal polarizations. The cross‐signs in (b, c) denote the two laterally displaced, cofocal spots at $\left(-x_{0},0,f\right)$ and $\left(x_{0},0,f\right)$. The black dot in the center denotes the center of the metalens. The scale bar for (b, c) is 2 μm.

Next, we focus on the polarization‐resolved photodetection functionality of our LPBS metalensed SNSPDs by quantifying the co‐ and cross‐detection probabilities. Here, the co‐detection probability $\left(P_{x\to x}\right)$ is defined as the probability of photodetection by an LPBS metalensed SNSPD with its nanowire oriented along the x‐direction when illuminated by x‐polarized light. On the other hand, the cross‐detection probability $\left(P_{x\to y}\right)$ is the probability of photodetection by the nanowire oriented along the y‐direction when illuminated by x‐polarized light. These quantities are computed in the FDTD solver (Tidy3D by Flexcompute Inc.) [64] from Equation (1) by integrating the ohmic‐loss density over the nanowire volume and normalizing to unit incident power. Figure 4a plots the co‐ and cross‐detection probabilities when the LPBS metalens is illuminated with x‐ and y‐polarized light as a function of the distance (center‐to‐center) $d=2x_{0}$ between the detectors. We see that, for both polarizations and for d> 10 μm, the cross‐detection events are negligible, varying between 1–2 %. For ideal polarization‐resolved photodetection, the cross‐detection probability should be zero, and the response should be the same for x‐ and y‐polarized light. Another metric for quantifying the performance of our LPBS metalensed SNSPDs is the cross talk for both polarizations defined [65]:

(4) $${XT}_{x}\;\left[dB\right]=10\log_{10}\;\left(\frac{P_{x\to y}}{P_{x\to x}}\right),$$

and analogous for y. At d = 10 μm, we obtained ${XT}_{x}$ = −15.06 dB and ${XT}_{y}$ = −18.27 dB. Our device's response is not identical for x‐ and y‐polarized light, and this deviation from the ideal behavior may be due to differences in the focusing performance of the metalens for x‐ and y‐polarized light, which are commonly observed in dielectric metalenses [66, 67, 68] (see Supporting Information S1: Figure S5b,c for linearly polarized light). Figure 4b,c shows intensity maps of the LPBS metalensed SNSPDs for x‐polarized and y‐polarized light when the two detectors are separated by d = 10 μm. The top panels display x‐z cross‐sections, and bottom panels show focal plane x‐y maps at $z=f_{s}$. Analogous results for left‐circularly polarized light are shown in Supporting Information S1: Figures S5d and S6a,b.

**FIGURE 4:**
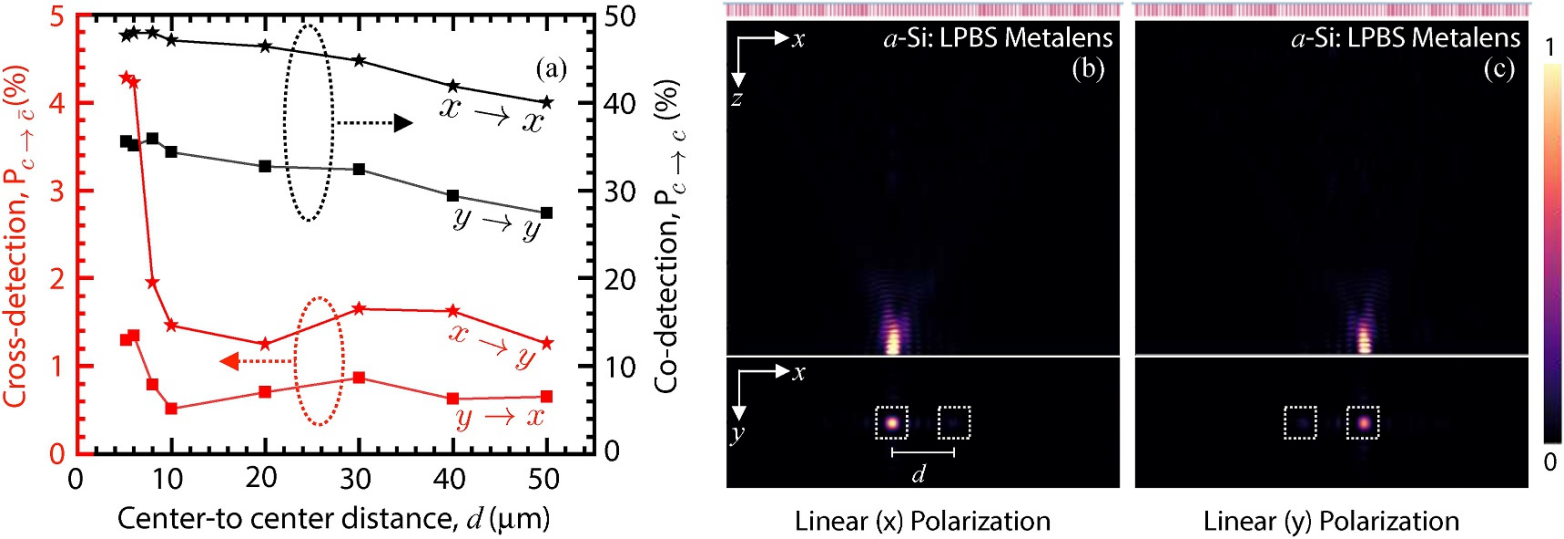
Polarization‐resolved photodetection using LPBS metalensed SNSPDs. (a) Plot of the co‐detection probability $\left(P_{c\to c}\right)$ and cross‐detection probability $\left(P_{c\to \bar{c}}\right)$ versus the center‐to‐center distance $d=2x_{0}$ between the detectors. (b, c) Field intensity maps of the LPBS metalensed SNSPD at d=10μm for x‐linear and y‐linear inputs, respectively. Top: meridional x‐z slices showing off‐axis focusing to $\pm x_{0}$. Bottom: focal plane x‐y maps at $z=f_{s}$.

## 4 Discussion and Conclusions

A viable way to precisely align a metalens at the required distance from the SNSPD within an optical cryostat is to mount the metalens on a z‐axis nanopositioner, whereas the SNSPD is mounted on an x‐y nanopositioner. This modular approach provides the flexibility to actively correct for any lateral shift (out‐of‐focus) in the position of either the metalens or the SNSPD when the cryostat is cooled to 4 K. Such corrections are routinely performed in an optical cryostat equipped with cryogenic objective lenses [69, 70, 71], offering a distinct advantage for a single‐element, single‐pixel SNSPD. However, scaling up this approach to an array of SNSPD pixels without compromising the enhancement factor may be challenging. Another approach is to fabricate the metalens directly on top of the SNSPDs, similar to microlenses [49], by incorporating a spacer layer. Here, the alignment can be achieved within the precision limits of the lithography tools. This integrated device would exhibit little to no lateral misalignment at 4 K, thereby maintaining its performance (see Supporting Information S1: Section 3, Figure S3a). However, it would involve complex nanofabrication, which could reduce the device yield. On the other hand, this approach offers a promising route to scaling up the device to multipixel SNSPDs, leading to the development of a single‐photon camera with high SDE while maintaining low reset time and jitter. More recently, plasmon‐enhanced superconducting photodetectors have been investigated to increase the photodetection efficiency. For instance, at 1550 nm, the SDE of an NbN nanowire reached up to 0.4% when coupled to silver nanoantennas [72]. On the other hand, the photon detection efficiency of an NbN microwire reached up to 98% for visible photons when integrated with nanocavities formed by arrays of silver nanocrystals [73].

In summary, we have proposed a long‐sought‐after solution for a miniaturized single‐element SNSPD with high SDE while preserving the benefits of a smaller active area. We achieved this by integrating a dielectric metalens on top of a miniaturized SNSPD, thereby increasing the SNSPD's effective light‐collection area. We show that under plane‐wave‐like illumination at 1550 nm, a moderately sized (60 μm × 60 μm) metalens can provide a ∼77‐fold increase in the SDE of an NbTiN SNSPD with an active area of 5 μm × 5 μm, compared with an identical reference SNSPD without a metalens. With a smaller active area, that is, a shorter nanowire, this integrated device would enable higher photon count rates while maintaining low reset time and jitter. Next, we designed a dielectric LPBS metalens integrated with a pair of mutually orthogonal NbTiN SNSPDs to achieve polarization‐resolved photodetection at 1550 nm. We achieved up to −18 dB of cross talk between detectors spaced 10 μm apart when illuminated with x‐polarized light, thereby enabling polarization‐resolved single‐photon detection at 1550 nm.

## Author Contributions

**Amir Targholizadeh:** investigation, methodology, validation, writing – review and editing, formal analysis, writing – original draft. **Grigoriy Y. Nikulin:** methodology, validation. **Pankaj K. Jha:** conceptualization, methodology, supervision, formal analysis, validation, investigation, writing – review and editing, writing – original draft.

## Funding

This work was partially supported by the Syracuse University Startup Funds.

## Conflicts of Interest

The authors declare no conflicts of interest.

## Supporting information


Supporting Information S1

## Data Availability

The data that support the findings of this study are available from the corresponding author upon reasonable request.
