# Supplementary material for: Cryogenic Dielectric Metasurface‐Integrated Superconducting Nanowire Single‐Photon Detectors
Source: Nanophotonics. 2026 Jun 28;15(13):e70172. doi: 10.1002/nap2.70172 (PMC13642542; doi:10.1002/nap2.70172)
Supplement: Supplementary file 1 [file NAP2-15-e70172-s001.pdf]

Supplemental Material for: Cryogenic Dielectric Metasurface-Integrated Superconducting Nanowire Single-Photon Detectors

Amir Targholizadeh,[1] Grigoriy Y. Nikulin,[1] and Pankaj K. Jha[1,2,*]

[1] *Quantum Technology Laboratory ⟨Q|T|L⟩, Department of Electrical Engineering and Computer Science, Syracuse University, Syracuse, NY 13244, USA*

[2] *Institute for Quantum & Information Sciences, Syracuse University, Syracuse, NY 13244, USA*

S1. CAVITY DESIGN

The detector cavity comprises, from top to bottom, air, a 8 nm NbTiN nanowire grating, a SiO_2 spacer of thickness t, and a 500 nm Au back reflector. The nanowire grating has 100 nm wire width and 50 % fill factor. The purpose of this section is to isolate the intrinsic absorptance of the optical stack under free-space excitation; system-level coupling from a focused beam is discussed in the main text. At normal incidence the Au mirror imposes an approximate π phase upon reflection. Choosing the spacer thickness near the quarter-wave condition $t \approx \lambda/(4n_{\mathrm{SiO_2}})$ establishes a Salisbury-screen interference that enhances the field within the lossy NbTiN while suppressing reflection, thereby maximizing absorptance.

We sweep t under plane-wave illumination and compute the absorptance localized in the NbTiN, as shown in Fig. S1(a). The optimum exceeds 92% for polarization parallel to the wire axis; the corresponding spacer thickness is $t_{\mathrm{opt}} = 233$ nm. With this thickness fixed, we rotate the incident linear polarization from 0° (parallel) to 90° (perpendicular) and observe a monotonic decline in absorptance, reaching approximately 45% at 90° [Fig. S1(b)]. This polarization dependence reflects the anisotropic current flow and form-birefringence of the nanowire grating together with the interference condition established by the spacer. Geometric and materials details of the detector stack and its integration are described in the main text; here we summarize the optical response relevant to absorber optimization under free-space coupling.

S2. METALENS DESIGN

All metalens simulations are performed at the telecom wavelength $\lambda = 1.55\,\mu$m. The clear aperture is $60\,\mu\text{m} \times 60\,\mu\text{m}$ and the target numerical aperture is NA = 0.5, corresponding to the maximum enhancement factor for NbTiN nanowires. The meta-atoms are cylindrical nanopillars of height $h = 0.85\,\mu$m arranged on a square lattice of pitch $p = 0.6\,\mu$m. To build the phase library, we compute the co-polarized propagation phase $\phi(r)$ and transmission $T(r)$ as functions of the pillar radius r using unit-cell simulations under normal incidence with periodic boundary conditions; this approach captures inter-cell coupling at the chosen pitch and yields the phase–amplitude relation used for layout, summarized in Fig. S2(a).

For fabrication robustness and layout simplicity, the continuous focusing phase is quantized into eight levels sepa-

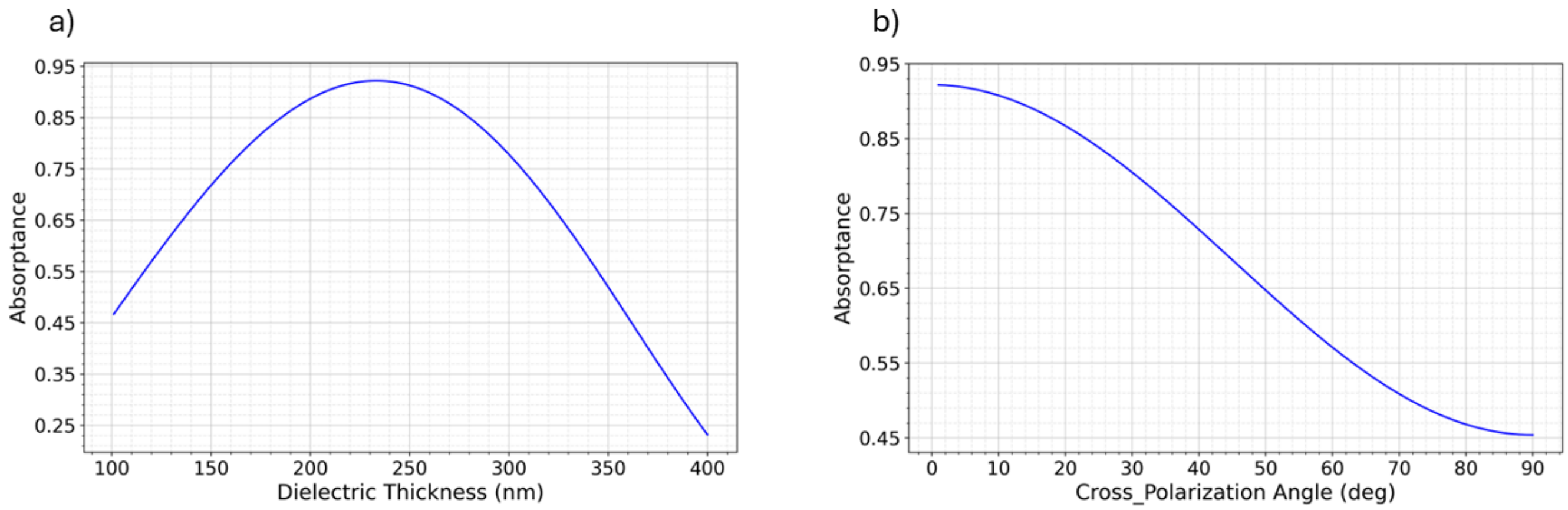

FIG. S1. Cavity optimization. (a) Absorptance versus SiO_2 spacer thickness for polarization parallel to the nanowire, exhibiting a peak exceeding 92%. (b) Polarization sweep at the fixed optimum thickness $t_{\mathrm{opt}} = 233\,\mathrm{nm}$, where the absorptance decreases to roughly 45% when the incident field is perpendicular to the wire. Simulations assume plane-wave illumination, 8 nm NbTiN with 100 nm wire width and 50 % fill factor, and a 500 nm Au back reflector.

rated by 45°. Cells whose continuous phase lies in the interval $(45°k, 45°(k+1)]$ are assigned to the mid-bin value $\phi_d = 22.5° + 45°k$ with $k = 0, \ldots, 7$. Radii are then selected from the library so that the realized phase $\phi(r)$ is as close as possible to ϕ_d at each lattice site. Focal performance is evaluated using the normalized intensity line cut at the focal plane along $y = 0$, shown in Fig. S2(b), from which the full width at half maximum (FWHM) is read from the half-maximum crossings.

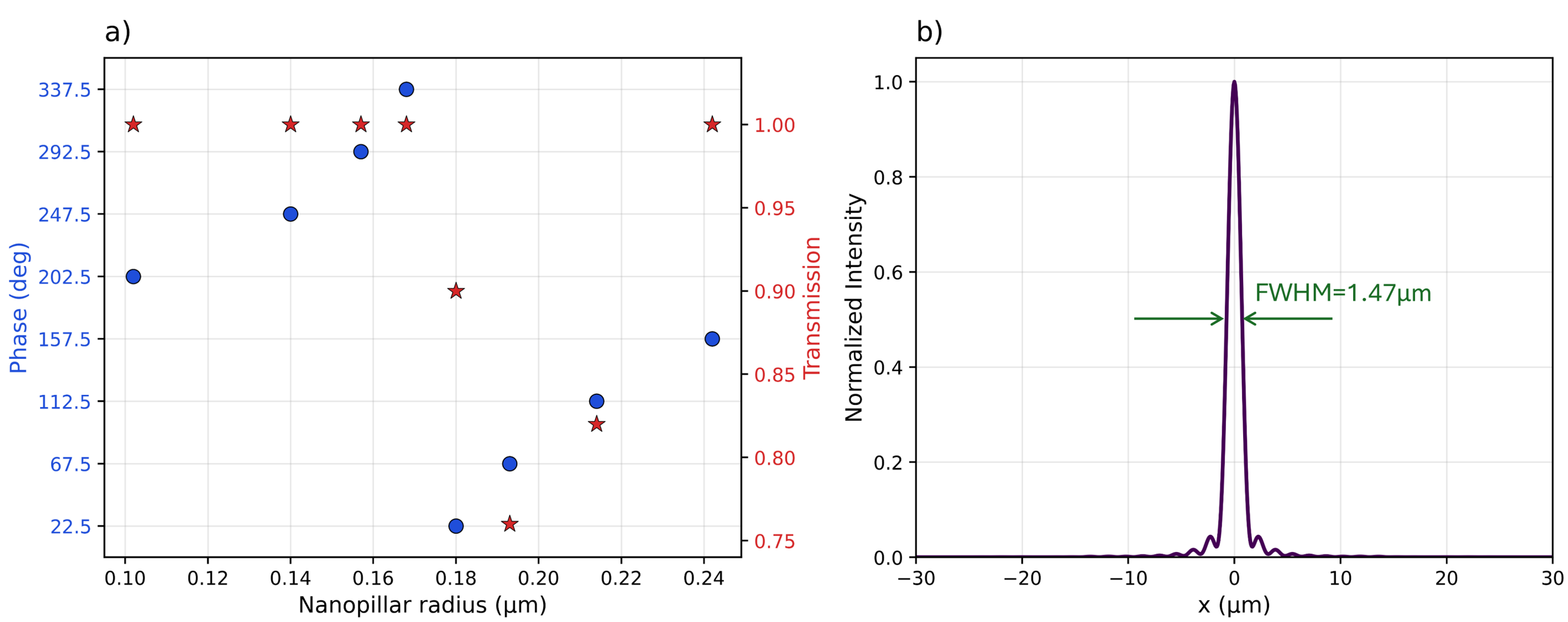

FIG. S2. Metalens characterization. (a) Phase (blue, left axis) and transmission (red, right axis) versus nanopillar radius extracted from FDTD simulations at pitch $p = 0.6\,\mu\mathrm{m}$ and height $h = 0.85\,\mu\mathrm{m}$. (b) Normalized intensity line cut at the focal plane ($NA = 0.5$) along y=0, with an example FWHM marker.

S3. FOCAL SHIFT AND DETECTOR DISPLACEMENT ANALYSIS

We investigate the focal shift between the designed and actual focal lengths and the sensitivity of our device to in-plane displacement. We first examine the focal shift. As discussed in the main text, the designed focal length f_{des} is typically larger than the simulated focal length f_{sim}, which we define as the axial location where the on-axis intensity $I(0,0,z)$ is maximized. We extract the fractional shift $\delta f/f = (f_{\mathrm{sim}} - f_{\mathrm{des}})/f_{\mathrm{des}}$ for different numerical apertures, plotted in Fig. S3(a), to provide a correction for accurate detector placement. To assess lateral tolerance, we then simulate the detector response as it is incrementally displaced along the x-axis at the focal plane. At each position, we record the enhancement factor [Fig. S3(b)]. The results show that the enhancement factor remains above 75 for displacements up to approximately 1.5 µm, indicating that the design is robust to typical fabrication and alignment imperfections.

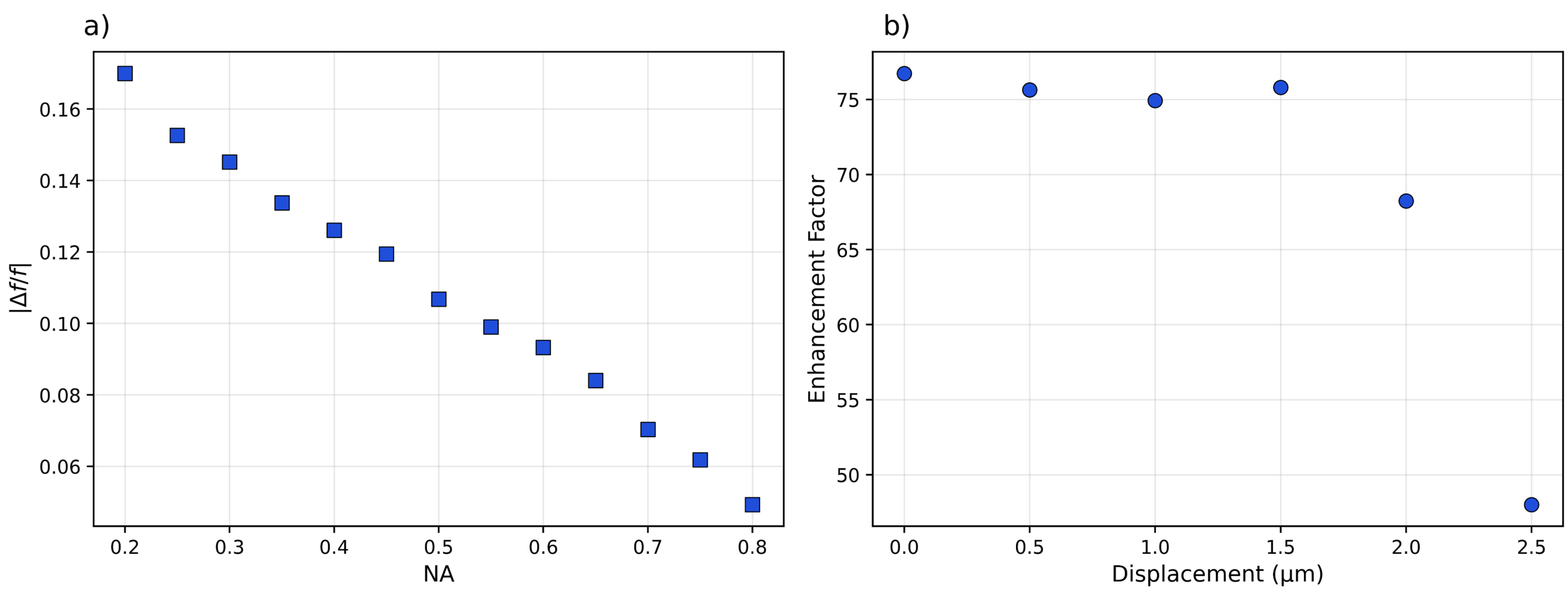

FIG. S3. Focal shift and misalignment robustness. (a) Fractional focal shift $|\delta f/f|$ as a function of numerical aperture. (b) Enhancement factor versus lateral detector displacement along the x-axis.

S4. COUPLING AND ABSORPTION EFFICIENCIES VERSUS NA

We separate the optical chain into two stages and define a coupling efficiency $\eta_{c,M} = P_{\mathrm{det}}/P_{\mathrm{in}}$ as the fraction of input power delivered to a 5 µm × 5 µm region centered on the design focus, with $P_{\mathrm{in}} = 1\,\mathrm{W}$ on the 60 µm × 60 µm aperture, and an absorption efficiency $\eta_{a,M} = P_{\mathrm{abs}}/P_{\mathrm{det}}$ as the fraction of detector-coupled power absorbed in the NbTiN, with $P_{\mathrm{abs}} = \frac{1}{2}\sigma \int |\mathbf{E}|^2\, dV$ integrated over the nanowire volume. We compute $\eta_{c,M}$ from a metalens-only simulation with no nanowires and $\eta_{a,M}$ from a separate simulation that includes the full detector stack.

The two efficiencies are plotted against numerical aperture in Fig. S4. A sweet spot emerges in the range NA ≈ 0.4–0.65, where $\eta_{c,M}$ and $\eta_{a,M}$ are simultaneously near their maxima at roughly 65% and 73–75%, respectively. We adopt

NA = 0.5 as our design value within this range.

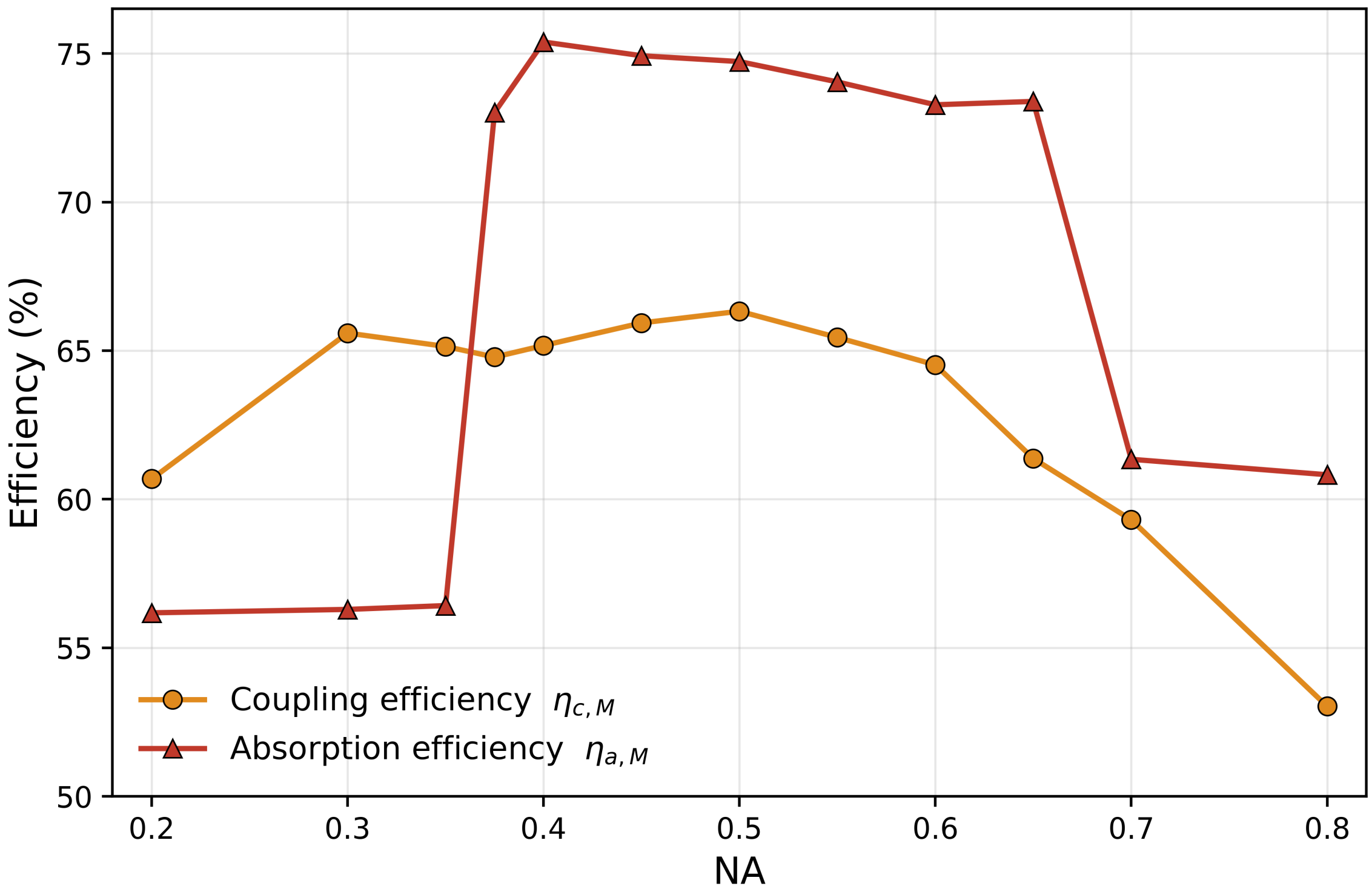

FIG. S4. Coupling and absorption efficiencies vs. NA. Coupling efficiency $\eta_{c,M} = P_{\text{det}}/P_{\text{in}}$ (orange circles) and absorption efficiency $\eta_{a,M} = P_{\text{abs}}/P_{\text{det}}$ (red triangles) as a function of numerical aperture. Both quantities are simultaneously high in the range NA$\approx$0.4–0.65, identifying a design sweet spot.

S5. LPBSM: LINEAR-POLARIZATION BEAM SPLITTER METALENS

The Linear-Polarization Beam Splitter Metalens (LPBSM) is a metasurface that routes orthogonal linear polarizations to distinct lateral foci, enabling simultaneous polarization discrimination at the focal plane. This section concerns the metasurface alone, without any nanowire detectors or coupling analysis. The LPBSM employs anisotropic nanobricks on a square lattice of period 0.5 µm and fixed height $h = 1.96$ µm. The lateral dimensions (ℓ_x, ℓ_y) govern two largely independent propagation phases for x- and y-polarized inputs, denoted $\phi_x(\ell_x, \ell_y)$ and $\phi_y(\ell_x, \ell_y)$.

To construct a reliable bivariate phase library, we first carried out a coarse sweep over a 20×20 grid of (ℓ_x, ℓ_y) pairs, totaling 400 unit-cell simulations, to sample the maps $(\ell_x, \ell_y) \mapsto (\phi_x, \phi_y)$ together with the associated transmissions. These coarse data were then interpolated to produce a continuous lookup that predicts (ℓ_x, ℓ_y) for arbitrary target phases. The metasurface design itself uses eight discrete phase levels for each polarization with 45° spacing, yielding $8 \times 8 = 64$ nominal nanobrick types. After assigning bricks via the interpolated lookup, we re-simulated the 64 chosen geometries and made small adjustments to (ℓ_x, ℓ_y) so that the realized phases matched the desired discrete targets as closely as possible. The final library of lateral dimensions is presented in Table I. The realized discrete phases for

x- and y-polarized inputs as a function of nanobrick index are shown in Fig. S5(a), and line cuts of the normalized focal intensity along $y = 0$ for x-polarized, y-polarized, and left-circularly polarized (LCP) illumination are shown in Fig. S5(b–d), with the FWHM values for the linear-polarization cases indicated on the plots.

TABLE S1. Final nanobrick library for the LPBSM. Lateral dimensions (ℓ_x, ℓ_y) in µm at height $h = 1.96\,\mu\text{m}$; eight discrete phase levels are used for each polarization.

y-pol \ x-pol	$\pi/8$	$3\pi/8$	$5\pi/8$	$7\pi/8$	$9\pi/8$	$11\pi/8$	$13\pi/8$	$15\pi/8$
$\pi/8$	(0.238, 0.238)	(0.248, 0.227)	(0.266, 0.224)	(0.298, 0.210)	(0.252, 0.369)	(0.337, 0.206)	(0.160, 0.270)	(0.195, 0.245)
$3\pi/8$	(0.220, 0.259)	(0.248, 0.248)	(0.259, 0.234)	(0.284, 0.231)	(0.248, 0.390)	(0.100, 0.369)	(0.153, 0.302)	(0.188, 0.291)
$5\pi/8$	(0.220, 0.270)	(0.234, 0.256)	(0.345, 0.345)	(0.386, 0.323)	(0.425, 0.302)	(0.100, 0.386)	(0.153, 0.309)	(0.188, 0.273)
$7\pi/8$	(0.210, 0.302)	(0.227, 0.294)	(0.425, 0.234)	(0.266, 0.266)	(0.241, 0.439)	(0.446, 0.319)	(0.149, 0.316)	(0.178, 0.319)
$9\pi/8$	(0.206, 0.323)	(0.227, 0.280)	(0.302, 0.425)	(0.450, 0.227)	(0.280, 0.280)	(0.294, 0.273)	(0.142, 0.386)	(0.171, 0.355)
$11\pi/8$	(0.206, 0.337)	(0.213, 0.333)	(0.386, 0.100)	(0.263, 0.298)	(0.351, 0.418)	(0.153, 0.153)	(0.210, 0.132)	(0.263, 0.118)
$13\pi/8$	(0.273, 0.160)	(0.298, 0.153)	(0.309, 0.153)	(0.355, 0.139)	(0.294, 0.245)	(0.128, 0.220)	(0.408, 0.404)	(0.224, 0.174)
$15\pi/8$	(0.248, 0.195)	(0.266, 0.192)	(0.287, 0.189)	(0.316, 0.178)	(0.355, 0.171)	(0.118, 0.263)	(0.174, 0.224)	(0.210, 0.210)

S6. LPBSM INTENSITY MAPS UNDER CIRCULAR POLARIZATION

We characterize the focusing behavior of the LPBSM under LCP illumination by mapping the field-intensity distribution $|E|^2$ in two complementary planes: a side view in the xz plane through the optical axis [Fig. S6(a)], and the focal-plane xy map at the simulated focal length [Fig. S6(b)]. The simulation uses an LCP plane-wave source incident on the LPBSM aperture, with all other simulation parameters identical to the linear-polarization cases.

The intensity maps in Fig. S6 reveal a pair of focal spots of comparable peak intensity, symmetric about the optical axis at the lateral positions $(\pm d, 0)$ associated with the two polarization channels. The side view confirms that both foci form simultaneously at the design focal plane. This behavior demonstrates that the LPBSM operates as a polarization-resolving element under circularly polarized illumination, separating the two orthogonal linear components into distinct spatial channels rather than introducing polarization-dependent loss.

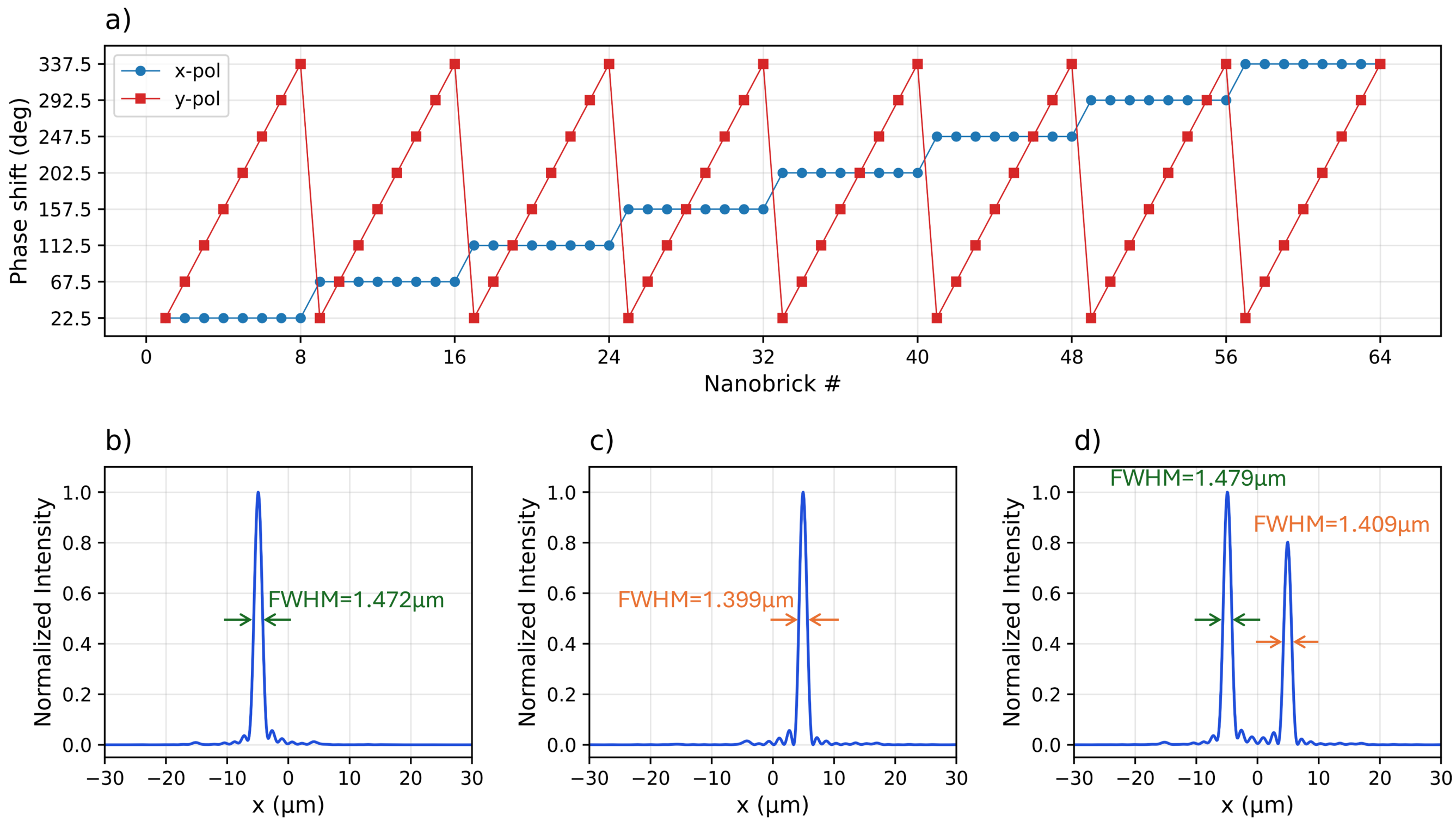

FIG. S5. LPBSM performance. (a) Realized discrete phases for x- and y-polarized inputs versus nanobrick index using eight phase levels. (b-d) Normalized focal-plane line cuts along y=0 for x-polarized, y-polarized, and left-circularly polarized (LCP) illumination, with FWHM values marked for the linear cases.

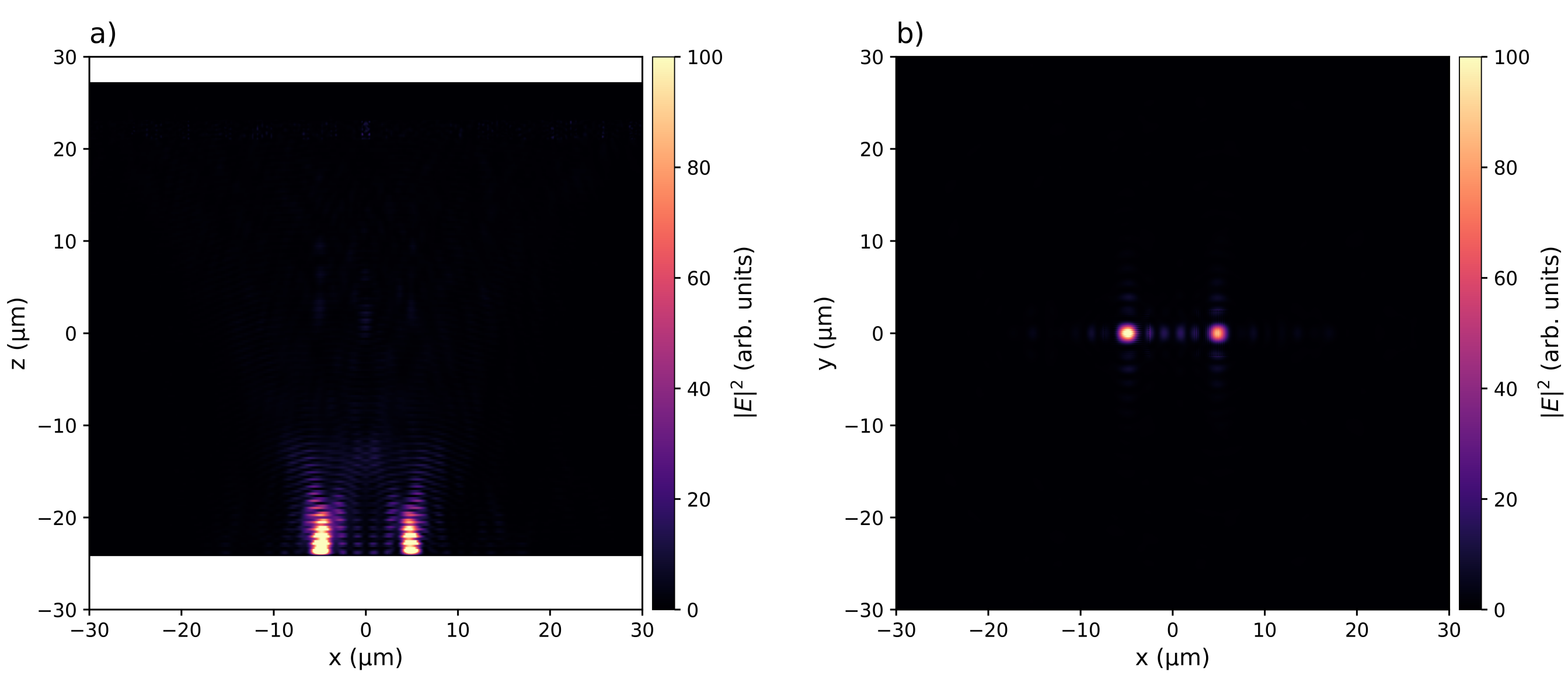

FIG. S6. LPBSM intensity maps under LCP illumination. (a) $|E|^2$ in the xz plane. (b) $|E|^2$ in the focal plane. Two foci of comparable intensity form at $(\pm d, 0)$, demonstrating polarization-resolving operation under circularly polarized illumination.
